# Top-Down Nanofabrication and Characterization of 20 nm Silicon Nanowires for Biosensing Applications

**DOI:** 10.1371/journal.pone.0152318

**Published:** 2016-03-29

**Authors:** M. Nuzaihan M. N, U. Hashim, M. K. Md Arshad, A. Rahim Ruslinda, S. F. A. Rahman, M. F. M. Fathil, Mohd. H. Ismail

**Affiliations:** 1 Institute of Nano Electronic Engineering, Universiti Malaysia Perlis, 01000 Kangar, Perlis, Malaysia; 2 School of Microelectronic Engineering, Universiti Malaysia Perlis (UniMAP), 02600 Pauh, Perlis, Malaysia; 3 Chemistry Department, Faculty of Science, Universiti Putra Malaysia, 43400 UPM Serdang, Selangor, Malaysia; Institute for Materials Science, GERMANY

## Abstract

A top-down nanofabrication approach is used to develop silicon nanowires from silicon-on-insulator (SOI) wafers and involves direct-write electron beam lithography (EBL), inductively coupled plasma-reactive ion etching (ICP-RIE) and a size reduction process. To achieve nanometer scale size, the crucial factors contributing to the EBL and size reduction processes are highlighted. The resulting silicon nanowires, which are 20 nm in width and 30 nm in height (with a triangular shape) and have a straight structure over the length of 400 μm, are fabricated precisely at the designed location on the device. The device is applied in biomolecule detection based on the changes in drain current (I_ds_), electrical resistance and conductance of the silicon nanowires upon hybridization to complementary target deoxyribonucleic acid (DNA). In this context, the scaled-down device exhibited superior performances in terms of good specificity and high sensitivity, with a limit of detection (LOD) of 10 fM, enables for efficient label-free, direct and higher-accuracy DNA molecules detection. Thus, this silicon nanowire can be used as an improved transducer and serves as novel biosensor for future biomedical diagnostic applications.

## Introduction

Silicon nanowires with nanometer-scale widths and micrometer-scale lengths are receiving considerable attention for sensor applications, and many research groups have demonstrated these materials’ promising nanotechnology and exciting potential for use in the future “Biosensing” era [1–7]. In particular, silicon nanowires offer interesting prospects for integration with complementary metal-oxide semiconductor (CMOS) [8–11] and lab-on-chip (LOC) technologies [12–13] as well as real-time [14–16], label-free [15–19] and high sensitivity sensing [1, 4, 15, 20–23]. The fabrication of silicon nanowires has been demonstrated by both bottom-up [3, 15, 24] and top-down [3, 8, 14, 22, 24–26] approaches.

The bottom-up approach usually uses metal-catalytic growth [4, 27]. However, there are several issues using this approach, as observed by Hsiao et al. [2]. In this report, difficulty in the exact positioning of nanowires on the device is the main issue, although metal contamination [2, 8] and the control of structural parameters [2] (i.e., randomly oriented growth and poor length and diameter size distributions) are other issues that need to be considered for the fabrication of nanowires.

In contrast, the top-down approach enables a more precise control of the geometry and a very accurate alignment with other structures on the device [14]. Better control of electrical properties can be achieved due to the very well defined widths and lengths of the silicon nanowires. Moreover, the top-down approach has higher yield and more reproducible results than does the bottom-up approach due to the maturity of the existing silicon fabrication and silicon-on-insulator (SOI) technologies.

Considering these advantages, we report a top-down nanofabrication of silicon nanowires using direct-write electron beam lithography (EBL). We describe the nanowire fabrication steps, such as sample preparation, pattern design, the EBL process, anisotropic etching using inductively coupled plasma-reactive ion etching (ICP-RIE) and the size reduction process. We highlight the important process parameters for EBL and size reduction for our nanometer-scale devices. Wire widths and heights as small as 20 nm and 30 nm, respectively, were fabricated on SOI substrates, and the nanowires successfully detected DNA molecules in a microfluidic environment by monitoring the changes in the current, resistance and conductance before and after DNA hybridization. These results prove that the silicon nanowires are promising for the detection of specific biomarkers and other targeted proteins.

## Methods and Materials

### 1. Top-down nanofabrication of silicon nanowires using EBL

**Fig 1:**
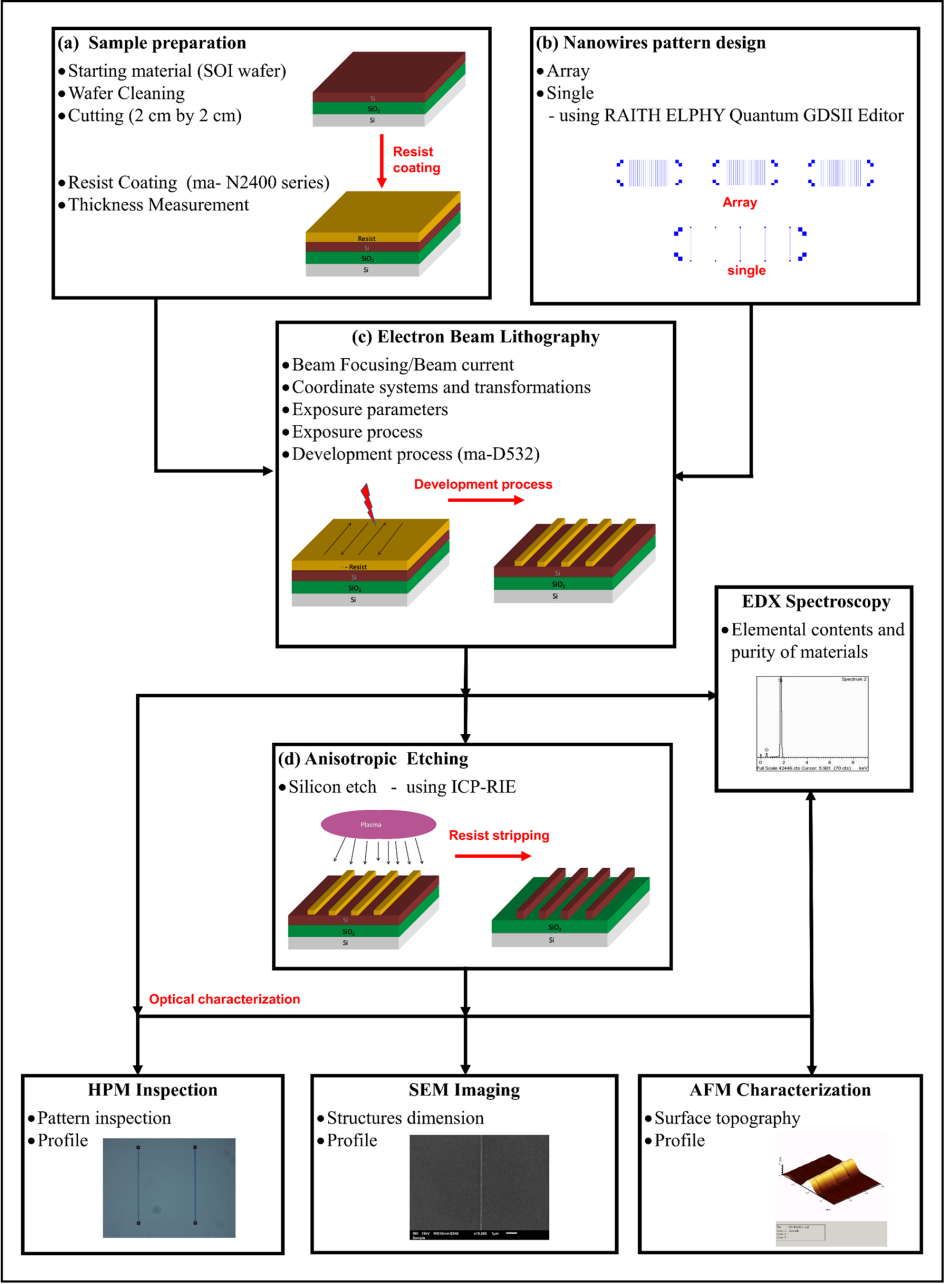
Top-down nanofabrication process steps of silicon nanowires using EBL.

The top-down nanofabrication process of silicon nanowires is briefly illustrated in Fig 1. Four key process steps are required to form the silicon nanowires: sample preparation, pattern design, EBL and anisotropic etching. Details of each process step are elaborated as follows.

#### a. Sample preparation

The silicon nanowires were fabricated from SOI (<100>) wafer (Soitec) with 200 nm of buried oxide (BOX) and a 50 nm p-type Boron-doped silicon top layer (resistivity: 8.5–11.5 Ω.cm with doping density of 10^15^ atoms.cm^-3^). First, the SOI wafer was cleaned using standard RCA 1 (mixing DI water: 5, ammonium hydroxide (27%): 1 and hydrogen peroxide (30%): 1) and RCA 2 (mixing DI water: 6, hydrochloric acid (30%): 1 and hydrogen peroxide (30%): 1) solutions to remove contaminants, followed by soaking in dilute hydrogen fluoride (HF) to remove the native oxide. All of the chemicals and solvents used in this cleaning process were purchased from Futurrex and Mallinckrodt Baker. After the cleaning process, the SOI wafer was cut into small pieces measuring 2 cm by 2 cm. Next, high-performance negative tone resists (ma-N2400 series were purchased from Microresist Technology GmbH) were spin coated on the sample and then dehydrated on a hotplate. The coated samples were then left for several minutes on a cooling plate to control the sample temperature for uniform resist characteristics. Detailed parameters of the resist coating process are summarized in Table 1. The ma-N2400 series resists are composed of a phenolic resin (novolak) as the polymeric bonding agent and an aromatic bisazide as the photoactive compound (PAC) dissolved in safer solvents, and they are very sensitive to electron beam radiation [28–30]. The advantages of the ma-N2400 series are its good thermal and etch stability. These resists can be developed without swelling in an alkaline aqueous developer and do not chemically modify the surface [28–30].

**Table 1:** ma-N2400 series coating process parameters.

Resist	Spin coat		Bake		Development		
	Speed (rpm)	Time (s)	Temp (°C)	Time (s)	Developer	Time (s)	Thickness (nm)
**ma-N 2403**	6000	30	90	60–90	Ma-D 532	30 ± 5	250
**ma-N 2405**	6000	30	90	60–90	Ma-D 532	45 ± 5	400

#### b. Nanowire pattern design

**Fig 2:**
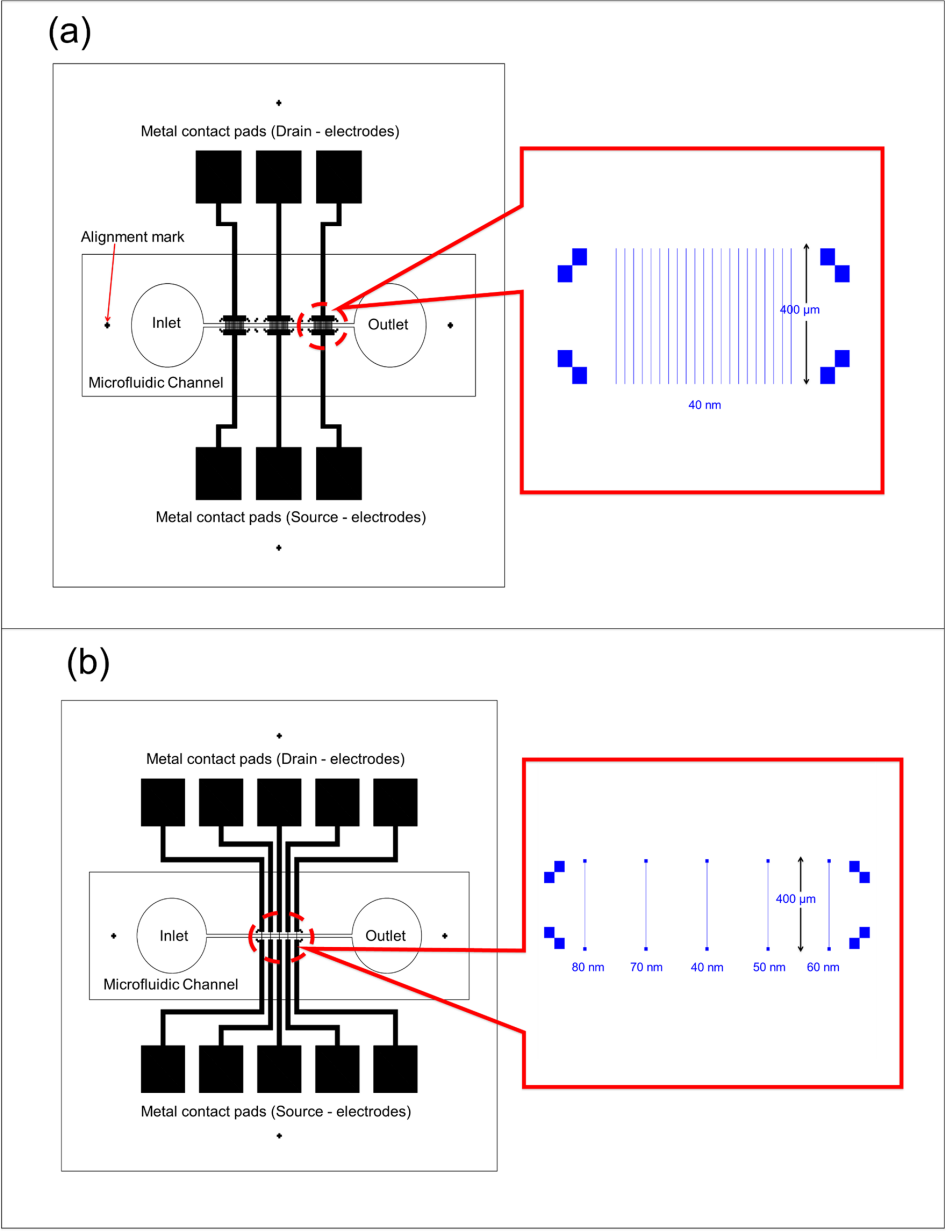
The overall design for silicon nanowires. (a) Array and (b) single, with the drain and source electrode pads.

The nanowire patterns were designed with various dimensions using RAITH ELPHY Quantum GDSII Editor developed by Raith GmbH. ELPHY Quantum is a universal lithography system that makes it possible to produce micro- and nanostructures by means of electron beam writing using a scanning electron microscope (SEM) [31], with pattern-placement accuracy below 20 nm [32]. Two type patterns were designed; one is an array of 20 identical nanowires with a 40-nm width and a 400-μm length (Fig 2(A)), and the other is an array of single nanowires with 5 different widths (40, 50, 60, 70 and 80 nm) and a 400-μm length (Fig 2(B)). For both designs, the 400-μm length was designed to ensure that the nanowires come into contact with the electrode pad in the subsequent fabrication process. In addition, both patterns were designed to increase the probability of adhesion or reaction of the analytes to the nanowire surface during testing [33].

#### c. Electron beam lithography (EBL)

The next step was the EBL exposure process. EBL was performed with JOEL JSM 6460LA SEM equipped with a Raith ELPHY Plus pattern generator. Detailed exposure process conditions for both resists are summarized in Table 2. After the EBL exposure process, the sample was left for 5 minutes before proceeding with the development process. Development was executed for various developing times using the ma-D 532 developer to determine whether the unexposed resist dissolved sufficiently. Developing times were varied from 15 to 45 seconds, depending on the thickness and type of resist. Then, the developed samples were rinsed in de-ionized water (DIW) for 5 minutes and blown dry with air. After rinsing, the samples were hard baked at 90°C for 60 to 90 seconds to improve the resist’s adhesion to the samples and its resistance to the anisotropic etching process. The developed samples were then characterized using high power microscopy (HPM), SEM (JEOL 6460), and atomic force microscopy (AFM).

**Table 2:** Exposure parameters.

Pattern Design	Array and Single nanowires
**Working Area**	600 μm X 600 μm
**Acc. Voltage**	20 keV
**Beam Current**	0.075 nA
**Dose Factor**	1.0
**Magnification**	400X–800X
**Electron Dose**	100 μC/cm^2^–200 μC/cm^2^

#### d. Anisotropic dry etching

Each developed sample was then loaded into a SAMCO ICP-RIE 10iP for anisotropic etch profile of silicon. This etcher can achieve anisotropic sidewall profiles in high aspect ratio openings. The resist pattern acted as a mask for the silicon etching [31] by protecting areas where a chemical reaction between the surface materials and reactive gases was not required. The ICP-RIE was performed under a pressure of 5 mTorr and a radio frequency (RF) power of 500 W using CF_4_ (30 sccm) and O_2_ plasma (28 sccm) for 1 minute. The resist pattern was stripped using acetone, which revealed silicon nanowires with a good anisotropic profile. The etch profile and feature sizes of the silicon nanowires were determined by SEM and AFM. In addition, energy-dispersive x-ray (EDX) was carried out to identify the elemental composition of nanowires after dry etching process.

### 2. Size reduction of silicon nanowires by thermal oxidation

**Fig 3:**
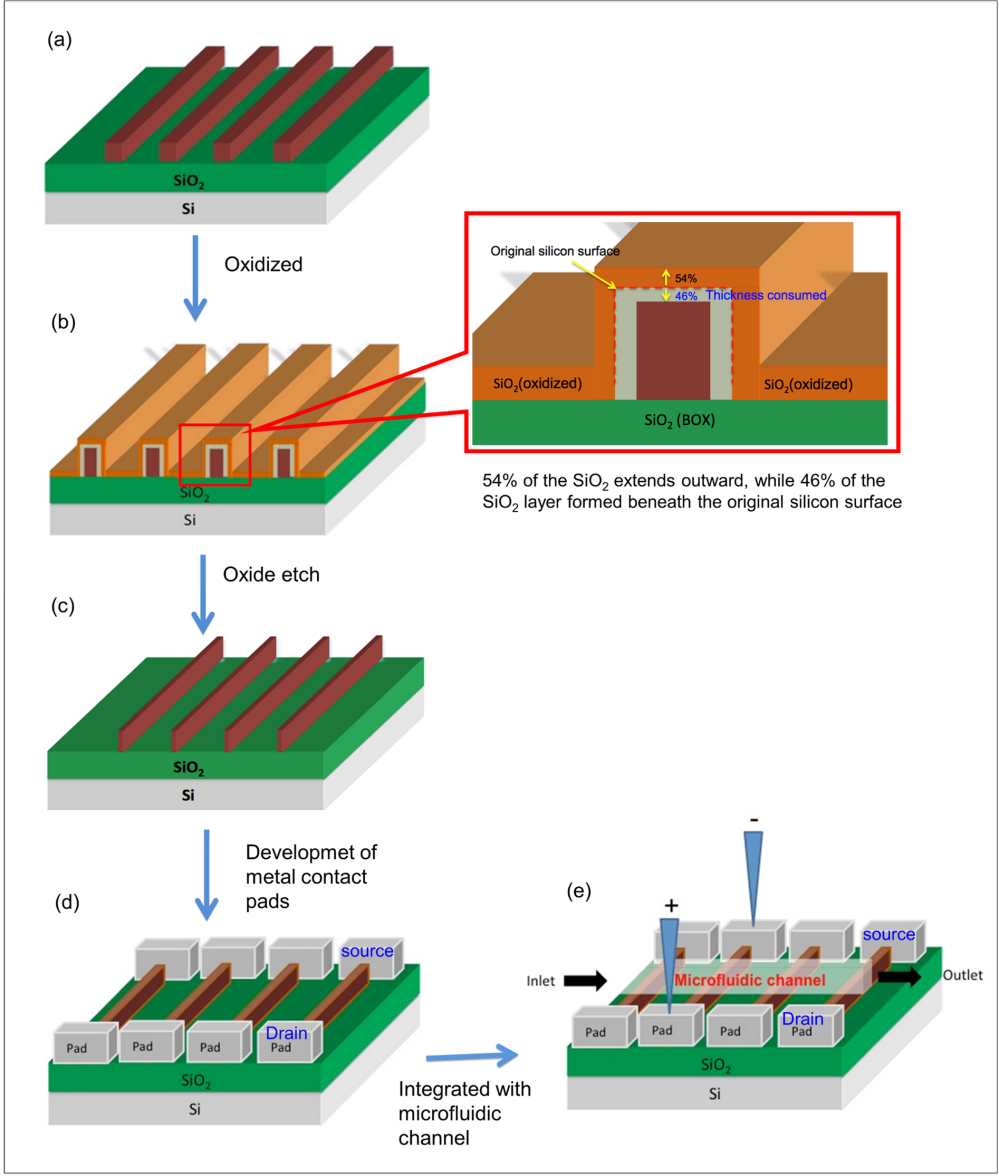
Process flow diagram. (a) Silicon nanowires produced by EBL and ICP-RIE, (b) size reduction by thermal oxidation, (c) BOE etching of the oxidized surface reduces the size of silicon nanowires, (d) silicon nanowires with the corresponding contact pads and (e) the sensor consisting of silicon nanowires integrated with the microfluidic channel.

The silicon nanowires were then oxidized in a dry O_2_ environment at 1000°C for 10–20 minutes, depending on the thickness of the silicon layer. To reduce the width of the silicon nanowires, the oxidized sample was dipped in buffered oxide etch (BOE) for 5 seconds to remove the SiO_2_ layer. The BOE soak time is crucial and thus needs to be optimized to avoid any removal of the buried oxide (BOX) and to maintain a layer isolating the electrodes from the substrate. To understand the size reduction process, Fig 3(A), 3(B) and 3(C) illustrate the process flow chart for size reduction by thermal oxidation. Fifty-four percent of the oxide formed outside the original silicon surface, and 46% of the oxide formed underneath it, all of which was removed during the BOE etching process. The aspect ratio was calculated by measuring the size difference in SEM and AFM images before and after the size reduction process.

### 3. Development of metal contact pads

After the fabrication of silicon nanowires using EBL and the size reduction process, metal contact pads were formed to allow electrical measurements on the fabricated silicon nanowires. In the device design, larger metal contact pads for the source (S) and drain (D) were connected to each end of the fabricated silicon nanowires. As shown in Fig 2(A) and 2(B), the array design allowed three pads with 20 nanowires per pad, while the single design allowed the five different size nanowires to be individually characterized. The pad mask was designed using AutoCAD and printed onto the chrome mask glass surface. Two different types of metal contact pads, 150 nm gold (Au) on 10 nm titanium (Ti) and 100 nm aluminum (Al), were developed using a lift-off method. First, the fabricated silicon nanowires were spin coated with negative resist (NR9-6000PY) at 3000 rpm for 25 seconds, followed by patterning with conventional lithography using a chrome pad mask. Subsequently, Au/Ti or Al was deposited onto the sample patterned with the negative resist using an Auto 306 thermal evaporator, followed by a resist stripping process for 3 minutes. Fig 3(D) shows the silicon nanowires with metal contacts at both ends (S and D) for further connection to the outer electronics. The rest of the silicon nanowire sensor was fabricated after the formation of metal contact pads.

### 4. Preparation of microfluidic channel

**Fig 4:**
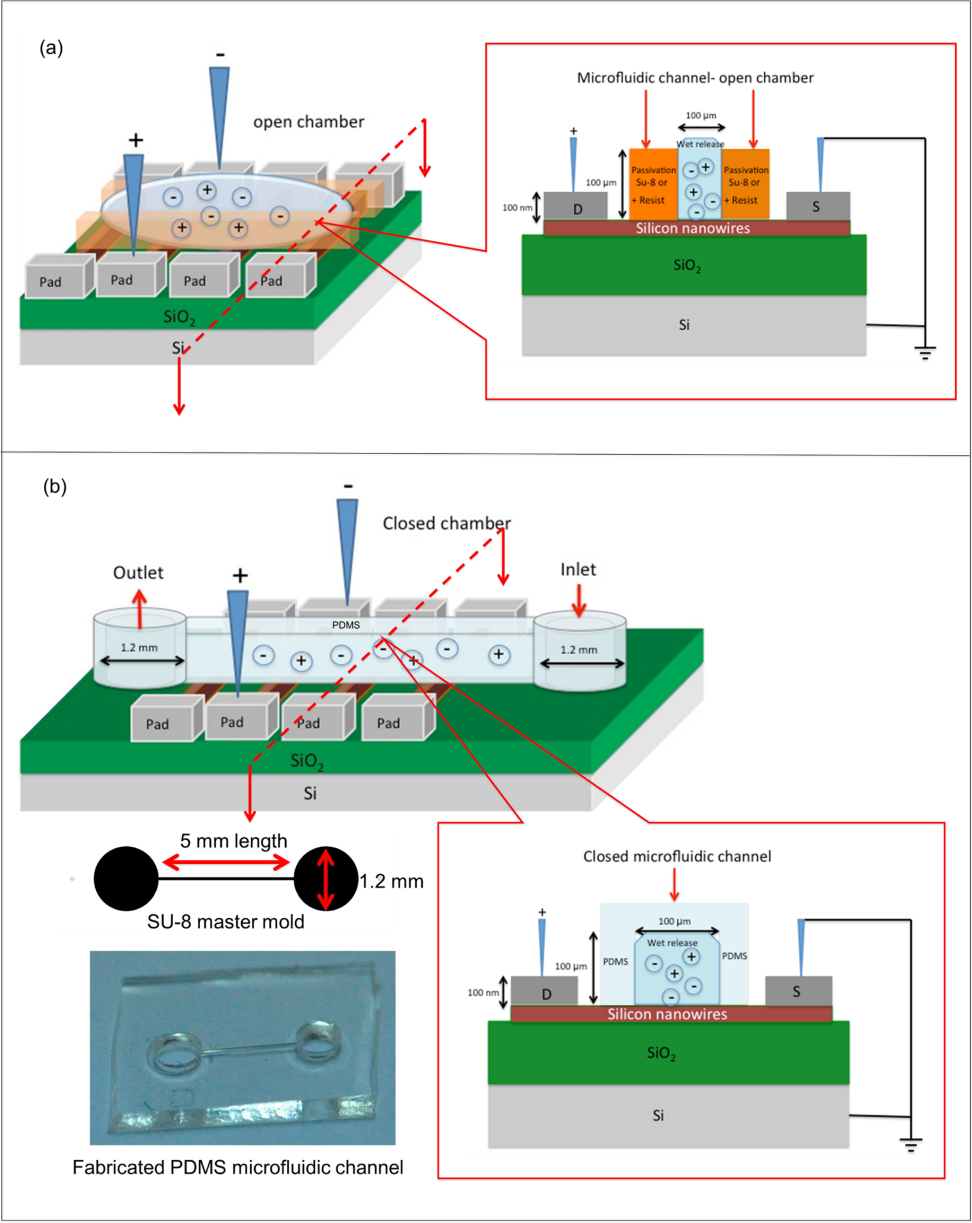
Schematic diagram of a cross-sectional view of the silicon nanowires integrated with the microfluidic channel. (a) Open chamber with a spin-coated resist (SU-8 or PR1-2000A) and (b) closed PDMS microfluidic channel.

The fabricated device was then integrated into a microfluidic channel. The microfluidic channel was fabricated on top of the silicon nanowires to allow solution to be directed over an active area of the silicon nanowires. Furthermore, the channel allows only a very small volume, which limits the flow and ensures the accurate detection of target DNA in the solution to be analyzed. The two main methods for microfluidic channel preparation are open chamber [7] and closed microfluidic channels [7], as shown in Fig 4(A) and 4(B), respectively. As seen in Fig 4(A), the open chamber microfluidic channel was lithographically patterned and developed on the fabricated device with a 100-μm-thick spin-coated negative resist (SU-8) or a positive photoresist (PR1-2000A). The dimensions of the open chamber microfluidic channel were 5 mm in length and 0.1 mm (100 μm) in width. For the closed microfluidic channel, a 0.1-mm (100-μm)-wide channel with 1.2-mm diameter inlet/outlet holes was fabricated using polydimethylsiloxane (PDMS) on a SU-8 master mold and then bonded to the fabricated silicon nanowires, as shown in Fig 4(B). Fabrication of the SU-8 master mold began with spin-coating SU-8 2010 series (MicroChem) at 300 rpm for 25 seconds on the cleaned silicon substrate surface and then baking at 95°C for 35 minutes. Next, the coated sample was exposed using conventional lithography for 240 seconds, followed by the development process. The PDMS pre-polymer was made from a 10:1 mass ratio mixture of a silicon elastomer base and a curing agent (Dow Corning’s Sylgard Elastomer 184); the mixture was vigorously stirred for 45 to 60 minutes to ensure a perfectly mixed pre-polymer and was subsequently poured on the master mold surface to generate a negative replica (i.e., an inverse structure) of the mold. The PDMS was then cured on the master mold at 70°C in an oven for 2 hours to remove all gas bubbles. The PDMS inverse structures were then mechanically peeled off the SU-8 master mold and cut to the final dimensions around the microfluidic channel as shown in Fig 4(B). Finally, the fabricated PDMS microfluidic channel and silicon nanowires were bonded together with an O_2_ plasma treatment at 300 mT of air pressure for 30 seconds.

### 5. Surface functionalization of silicon nanowires

**Fig 5:**
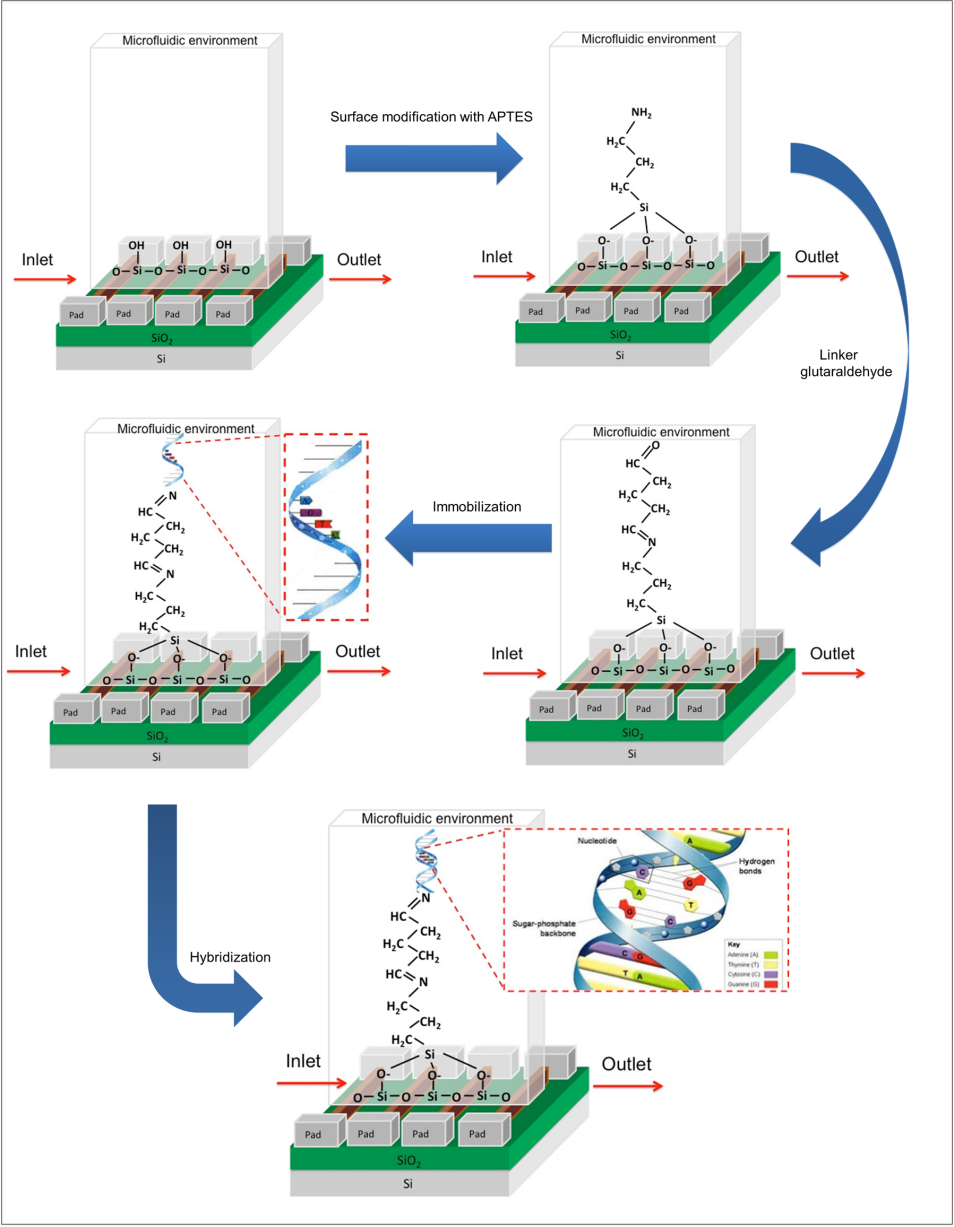
Schematic illustration of the surface functionalization. Surface modification by APTES and glutaraldehyde, DNA immobilization and DNA hybridization on the silicon nanowire surface.

Functionalization of the silicon nanowire surface is necessary to prepare a suitable platform for DNA detection. The three steps involved in the surface functionalization of silicon nanowires are surface modification, immobilization and hybridization, as shown in Fig 5.

#### a. Surface modification

Surface modification is a chemical process that favors the formation of the active groups that are necessary for the covalent immobilization of biomolecules on a surface. In this research, the surface modification began by cleaning the surface of nanowires using DI water and IPA, followed by drying for 5 minutes. Next, the silicon nanowires were immersed in 2% 3-aminopropyltriethoxysilane (APTES (v/v)) in a mixture of 95% ethanol and 5% water for 2 hours at room temperature to obtain surface-exposed amine groups on the surface of the nanowires [21]. Then, the samples were washed with ethanol 3 times to remove any unreacted APTES and dried on a hot plate at 120°C for 10 minutes. Next, the APTES-functionalized nanowires surface was immersed in a 2.5% glutaraldehyde (Sigma-Aldrich) with phosphate-buffered saline (PBS, pH 7.4) solution for 1 hour at room temperature. Glutaraldehyde was introduced as a linker to bind the amine-terminated APTES and present aldehyde groups on the surface. Subsequently, the samples were washed with PBS solution for 5 minutes to remove excess glutaraldehyde.

#### b. DNA immobilization

For the immobilization procedure, a 27-mer amine-terminated probe (5’-NH_2_-C_6_-AACAGCATATTGACGCTGGGAGAGACC-3’) (Integrated DNA Technologies, Inc.) was used to bind the available aldehyde groups on the silicon nanowire surface. This probe was diluted with PBS (pH 7.4) to a 10 μM DNA probe solution before immobilization on the APTES-functionalized nanowire surface. Next, the probe was injected into the microfluidic channel flowing through the silicon nanowire sensing area, and the sensor was incubated at room temperature for 4 hours. Then, any unbound probe was washed away with PBS 3 times.

#### c. DNA hybridization, de-hybridization and control

After immobilization, the 27-mer complementary target DNA (3’-TTGTCGTATAACTGC GACCCTCTCTGG-5’) (Integrated DNA Technologies (IDT), Inc.) was applied to hybridize the immobilized DNA on the nanowires. The sample was hybridized at room temperature overnight and then washed with PBS to remove the excess target DNA. The 27-mer complementary strand was diluted to various concentrations (10 fM to 10 μM) with PBS. To ensure successful hybridization, the electrical properties of the hybridization samples were then measured using I-V measurement. After the electrical measurements, the samples were washed with hot DI water at 90°C for 5 minutes to de-hybridize the complementary DNA pairs on the nanowires. To confirm the specificity of the sensor, the immobilized DNA that remained on the nanowires was hybridized with 27-mer (same length) non-complementary DNA (3’-CCTGTACCGGGTCTATGATTGTGTCTT-5’).

### 6. Optical and electrical characterization

The morphology and electrical properties of the silicon nanowires were then characterized. High power microscopy (HPM) (OLYMPUS-BX51) was used as the basic optical inspection tool for sample and device structures. Scanning electron microscopy (SEM) (JEOL JSM-6460LA) was used to determine the quality of the silicon nanowires, particularly the shape, diameter and uniformity. Energy-dispersive x-ray (EDX) was carried out to identify the elemental contents and purity of materials. Atomic force microscopy (AFM) (SPA400-SPI3800, Seiko Instruments Inc., Japan) was used to study the surface and 3D profile of the silicon nanowires. The electrical characterization was carried out to investigate the current-voltage (I-V), specificity and sensitivity of the silicon nanowire sensor using a KEITHLEY 6487 picoammeter/voltage source. A direct current (DC) voltage of 0–1 V was applied between the two metal contacts of silicon nanowire sensor to test the fabricated silicon nanowires, the amine-terminated APTES, DNA immobilization and hybridization. All measurements were performed at room temperature and under a microfluidic environment.

## Results and Discussion

### 1. Characteristics of the ma-N2400 series negative electron beam resist

**Fig 6:**
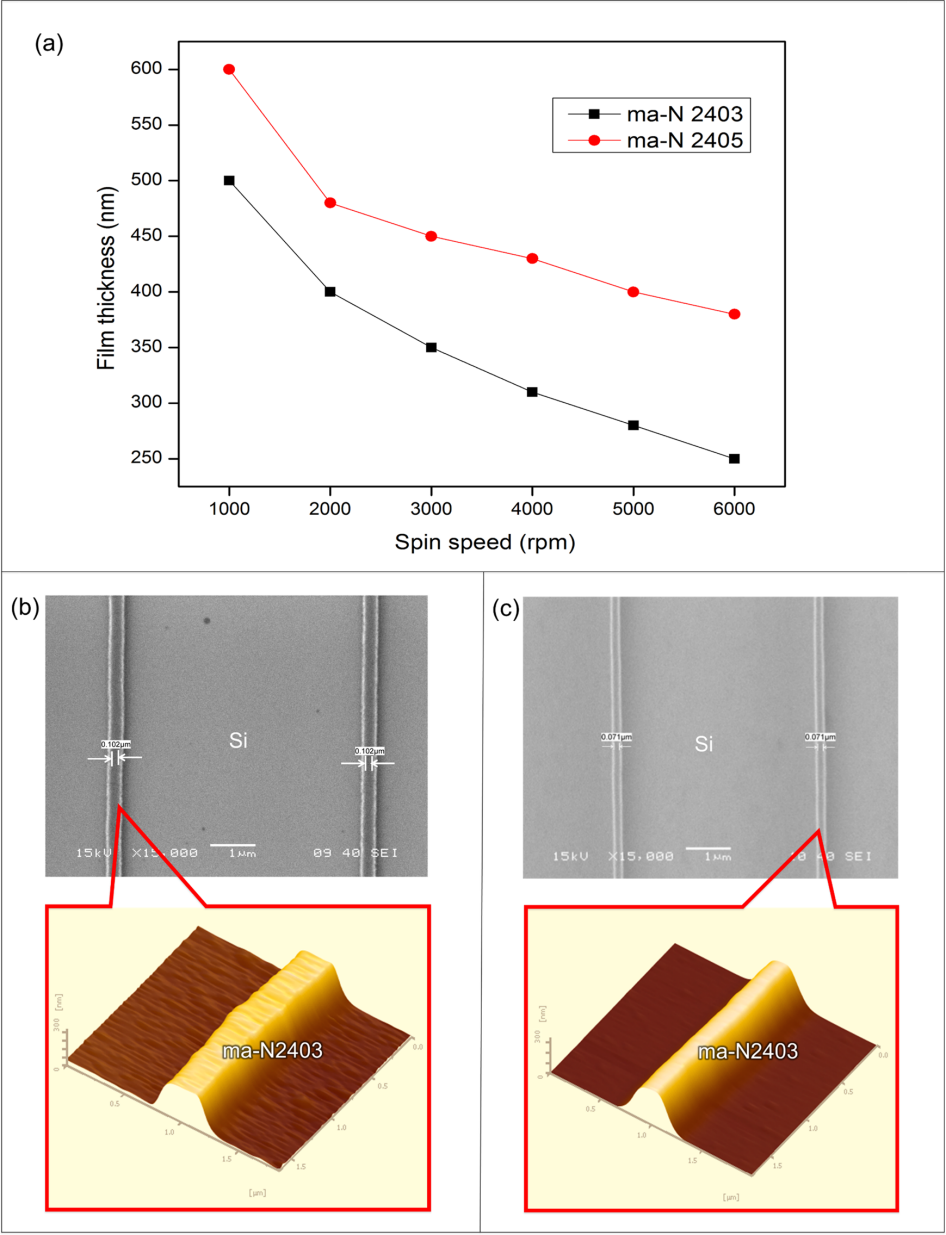
ma-N 2400 series resist characteristics. (a) The resist thickness curve decreasing with the spin speed (rpm), SEM and AFM images of resist pattern after development process (b) 100 nm width exposed with an electron dose of 200 μC/cm^2^ and (c) 70 nm width exposed with an electron dose of 150 μC/cm^2^.

An important aspect of this silicon nanowire fabrication method was the characteristics of negative electron beam resist. Fig 6(A) clearly shows that the resist film thickness is inversely proportional to the spin speed of the ma-N2400 series, whereby an increase in the spin speed process leads to a decrease in the resist thickness. This resist interacted with and adhered to the silicon surface, thus creating a thin electron beam resist layer. A thinner and more uniform electron beam resist layer is preferable to produce better resolution and good resistance to the plasma etching process, which are crucial for obtaining nanostructures with high aspect ratios [28]. The resulting ma-N2400 series resist film thicknesses were approximately 250–400 nm at maximum spin speed of 6000 rpm for 30 seconds, as shown in Fig 6(A). This is an optimal resist thickness for exposure parameters described in Table 2. The ma-N2403 is thinner than the ma-N2405 because of its different viscosity and density. To investigate the effect of dose on the feature size (width), the ma-N2403 e-beam resist coatings (250 nm) were exposed through the nanowire patterns with different electron doses, with the accelerating voltage and developing time kept constant at 20 keV and 40 seconds, respectively. Different feature sizes of e-beam resist have different dose requirements [34]. Fig 6(B) and 6(C) show SEM and AFM images of developed resist patterns with widths of approximately 100 nm and approximately 70 nm exposed with electron doses of 200 μC/cm^2^ and 150 μC/cm^2^, respectively. Fig 6(B) and 6(C) show that the feature size is very sensitive to electron dose value. As the dose increases, more electrons are transferred to the coated resist and the feature size become thicker [35]. This is in good agreement with the findings reported by Grigorescu et al. [36], who noted that the feature size increases with increasing electron doses. The high-resolution resist pattern with a thickness of 250 nm and a width of approximately 70 nm (Fig 6(C)) was later used for the silicon etch (dry) process by RIE to form the silicon nanowires. In addition to its excellent resolution capability, the ma-N2400 series resists are simple resists with easy process control and are considerably more resistant to dry and wet etching.

### 2. Morphology of the silicon nanowires

**Fig 7:**
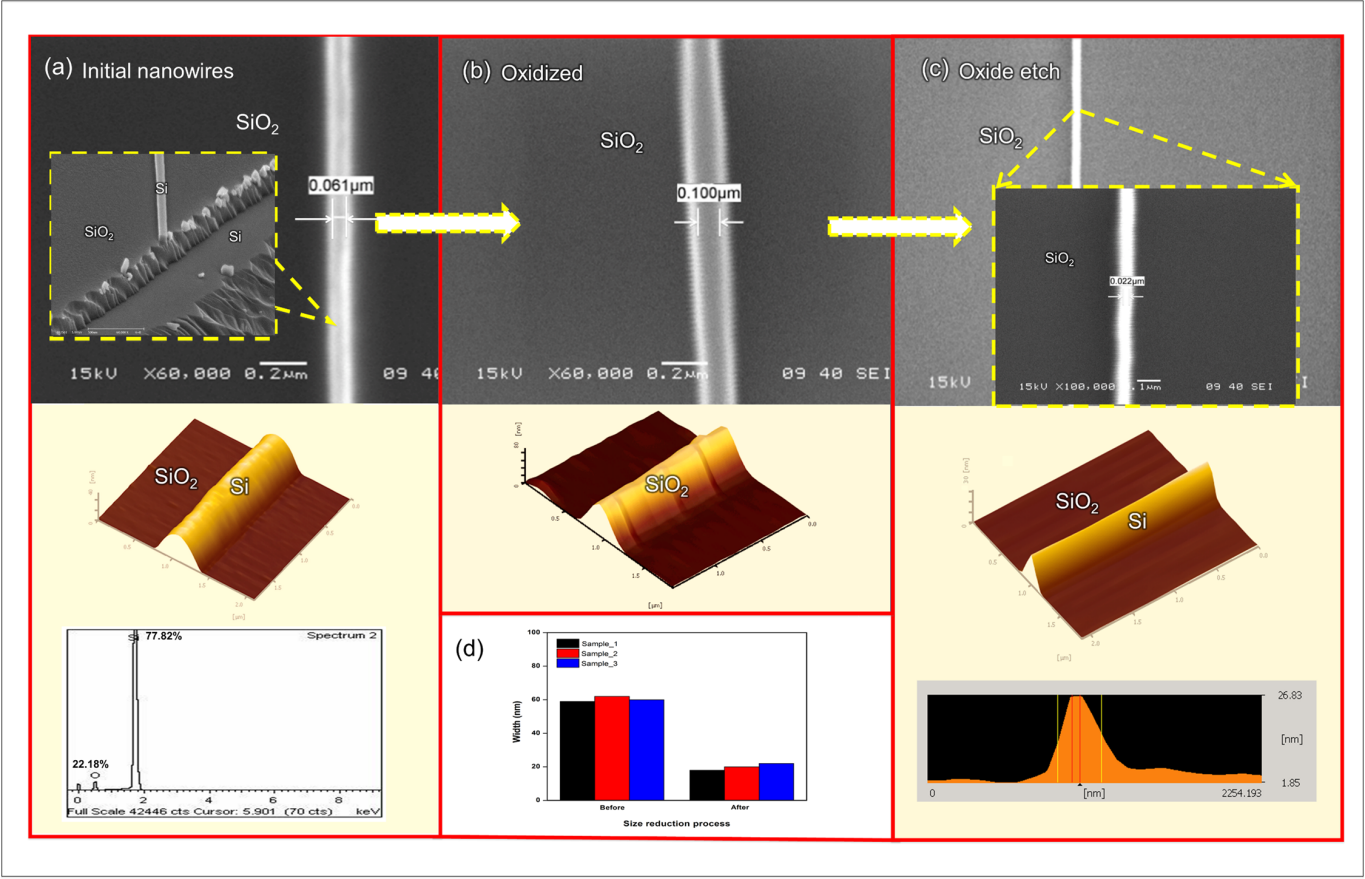
SEM and AFM images of the silicon nanowires. (a) 60 nm width and 50 nm height. The inset is the cross-section of the silicon nanowires. EDX spectrum shows the purity of silicon (Si) on the nanowire. (b) The silicon nanowires were oxidized to a width of 100 nm and then (c) etched in BOE to remove the SiO_2_, thus producing 20-nm-wide and 30-nm-high silicon nanowires. The inset is the SEM image of silicon nanowires at 15 keV with 100 000 magnification. (d) The size (width) of silicon nanowire for three samples (before and after size reduction process).

A top view SEM image of the silicon nanowires after the dry etch process is shown in Fig 7(A). The results indicate that the silicon nanowires were formed with good uniformity, high resolution and good pattern placement. The width of the silicon nanowires is approximately 60 nm. The cross-sectional SEM image shown in the Fig 7(A) inset further revealed that these nanowires exhibit clear rectangular cross sections, though the AFM image shows the profile features to be approximately 60 nm in width and 50 nm in height. Compared with the resist pattern in Fig 6(C), the silicon nanowires reduced in width by approximately 10 nm after the dry etch process. The results show that dry etching process produced a silicon nanowire with more surface roughness and greater width, which is in agreement with the findings of Enami et al. [37]. The size reduction process is the major focus of this research. To achieve the smallest possible width of silicon nanowires with good aspect ratios, dry oxidation and wet etching (BOE) are preferred [21, 38–40]. The width of the silicon nanowires could be precisely controlled by controlling the etching time and using self-limiting oxidation [21]. The 60-nm silicon nanowires were dry oxidized and first expanded to approximately 100 nm (Fig 7(B)). Then, the SiO_2_ was etched away, which resulted in a final width of 20 nm (Fig 7(C)). The AFM image in Fig 7(C) shows the morphology and a profile of approximately 20 nm in width and 30 nm in height. These images prove that 20 nm (46%) of the grown oxide extends inward and that 25 nm (54%) of the grown oxide extends outward from the original dimensions of the silicon nanowires. The aspect ratio h/w can be calculated using Eq (1), as shown below:

$$\frac{h}{w}=\frac{h_{initial}-c}{w_{initial}-2c}$$ (1)

where *h* is the height, *w* is the width and *c* is reduction value. The aspect ratio was calculated to be 1.5 when the observed values of *h* and *w* were 30 nm and 20 nm, respectively. The AFM results demonstrate that the top width of the silicon nanowires is smaller than its base width due to the limitations of the AFM physical probe, which is not ideally sharp. As a consequence, the AFM image reflects not the true sample topography but the interaction between the silicon nanowire and probe [41]. Hence, we have also demonstrated the accuracy and repeatability of measurements as shown in the inset of Fig 7(D). Three samples from each step (before and after reduction process) were used in the measurement test using AFM. The results show that the samples have good repeatability and very high accuracy. In addition, EDX was carried out to identify the elemental contents and purity of the nanowire. The EDX spectrum of the fabricated silicon nanowire is presented in Fig 7(A). The amount of silicon (Si) and oxygen (O) elements were achieved around 77.82% and 22.18% from the total percentage weight, respectively. There is a strong Si peak at 1.8 keV together with the O peak at 0.5 keV in the spotted nanowire, indicating that was silicon nanowire successfully fabricated.

### 3. Electrical characterization of fabricated silicon nanowires

**Fig 8:**
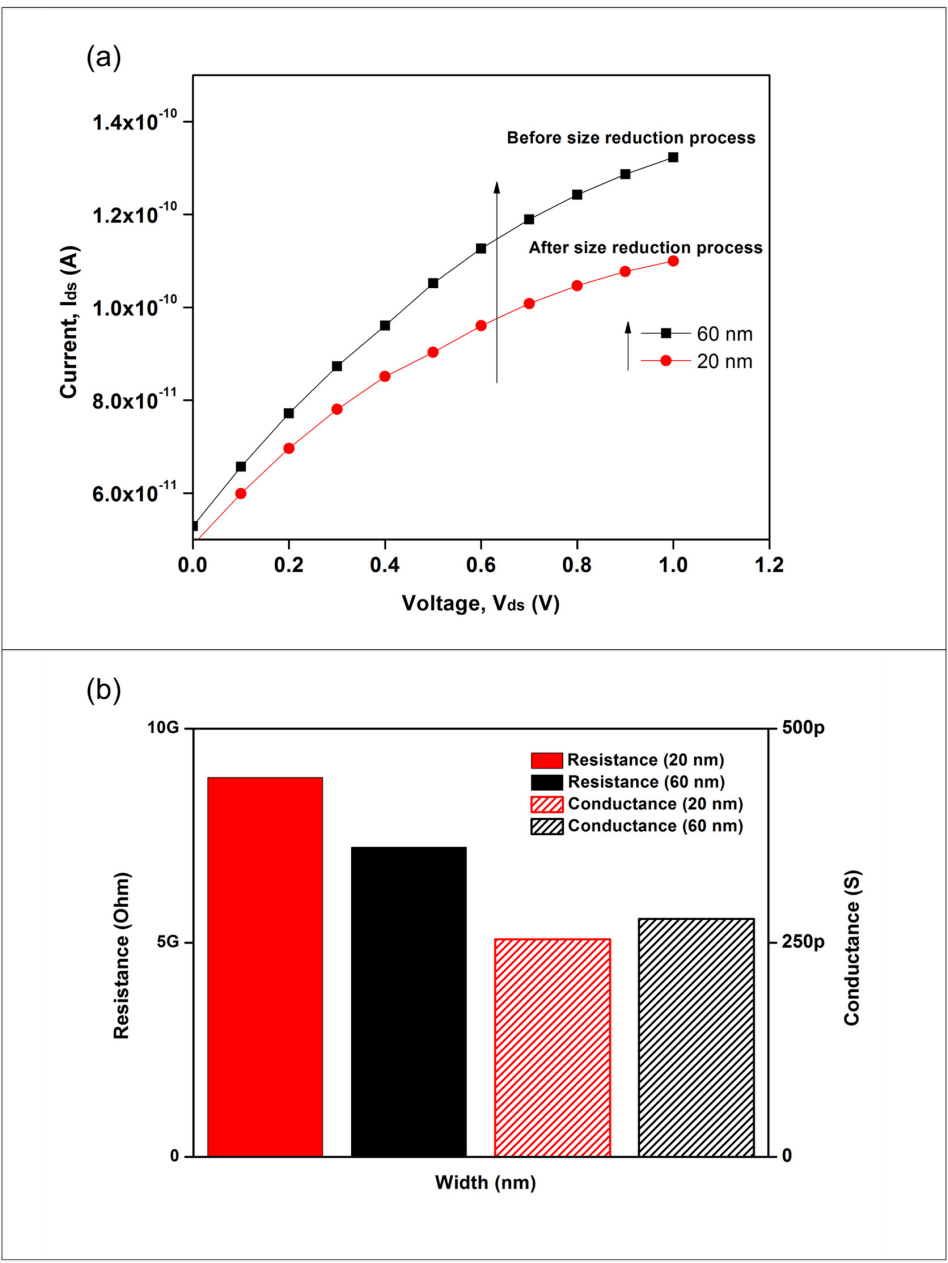
Electrical properties of silicon nanowires. (a) I_ds_-V_ds_ characteristic shows p-type ohmic behavior and (b) the resistance and conductance histograms for 20 nm and 60 nm nanowires. The arrow highlights the direction from low to high width of the silicon nanowires.

The quality of the silicon nanowires was further characterized electrically by measuring the current-voltage (I-V) characteristic between the source (S) and drain (D) electrodes before introducing DNA detection to the silicon nanowires. The I_ds_-V_ds_ characterization of the silicon nanowires after each stage (before and after size reduction process) was plotted as shown in Fig 8(A). The device exhibited an almost linear relation between the current and voltage (i.e., ohmic behavior). After the size reduction process (width = 20 nm), the drain current (I_ds_) was much lower than the I_ds_ measured before the size reduction process (width = 60 nm). At V_ds_ = 1 V, the I_ds_ value dropped by 23 pA, from 132 pA to 109 pA. The decrease was caused by the increase in resistance at the silicon nanowires after the size reduction process. Fig 8(B) shows that the average resistance values of silicon nanowires for 20 nm and 60 nm were 8.9 GΩ, and 7.2 GΩ, respectively. The resistance of the wire is inversely proportional to its width. The results indicate that the lightly doped silicon nanowires (10^15^ atoms.cm^-3^) produced high resistance and sensitivity due to the pronounced electromagnetic interference [17], which is important for biomedical applications. In addition, the electrical conductance for 20 nm and 60 nm was 0.25 nS and 0.27 nS, respectively. From Fig 8(B), the smaller feature size nanowire (20 nm) has lower conductance than does the bigger nanowire (60 nm) due to the large surface effects of smaller wire [14]. The electrical measurements confirmed that this top-down nanofabrication process produces high-quality silicon nanowires with great potential for further development.

### 4. Specificity and sensitivity of DNA detection by the 20 nm silicon nanowires

**Fig 9:**
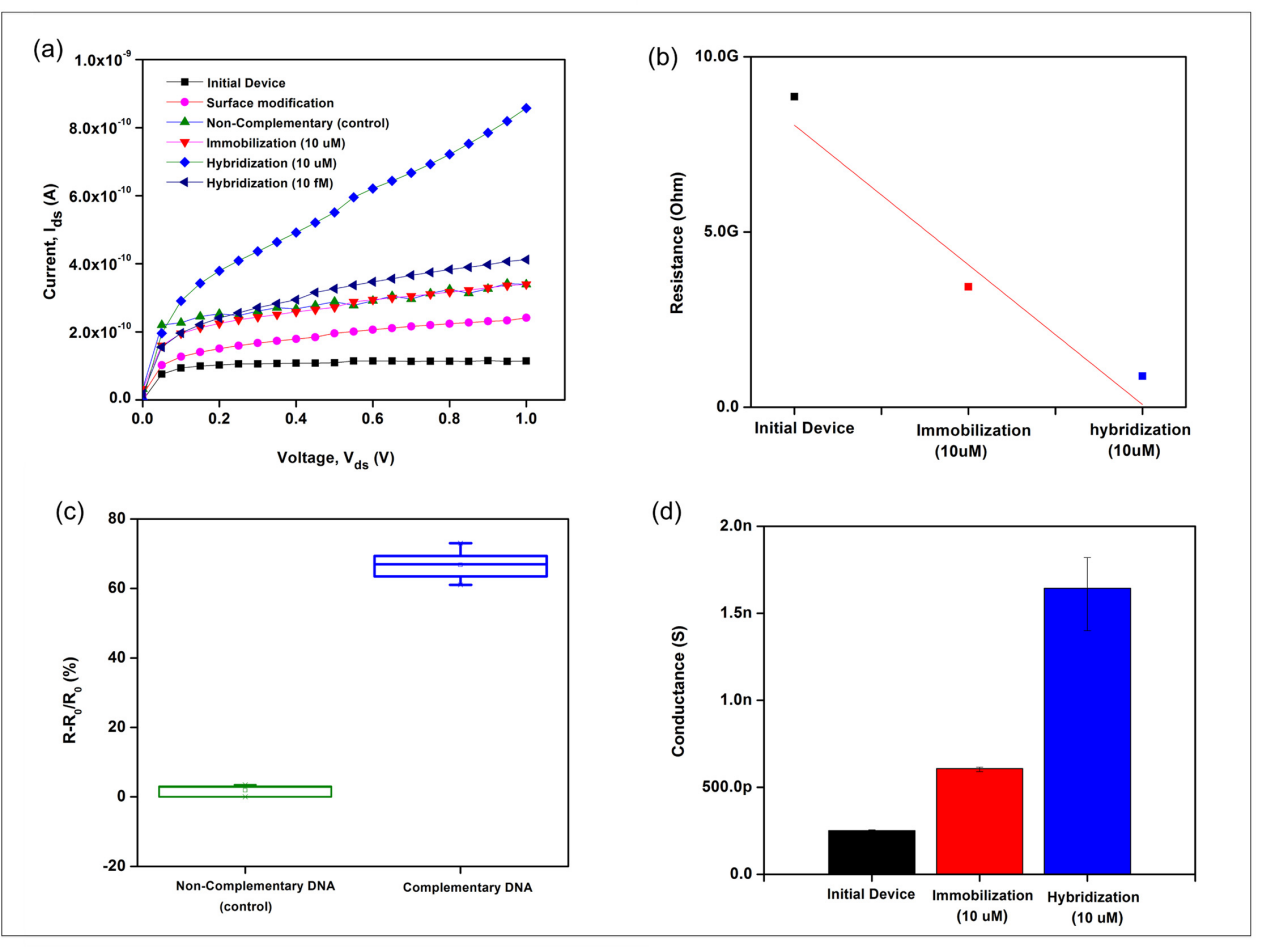
Silicon nanowire biosensor. (a) I_ds_-V_ds_ characteristic, (b) the average resistance values after various surface functionalizations, (c) hybridization specificity demonstrated by the response (i.e., resistance change) to the complementary and non-complementary DNA sequences and (d) the electrical conductance by different steps of surface functionalization.

To evaluate the specificity and sensitivity of DNA molecule detection, a 20-nm silicon nanowire that was perfectly aligned and integrated with the open chamber microfluidic channel was developed, as illustrated in Fig 4(A). To ensure that the target DNA was identified precisely and accurately, an amine-based method was used in this research. The large amount of SiOH (silanol) groups with excellent proton donors (H^+^) and acceptors (SiO^−^) [42] on the silicon nanowires/SiO_2_ surface (i.e., the native oxide) was reacted with an APTES solution to obtain surface-exposed–NH_2_ (amine) groups for biomolecule immobilization with the help of glutaraldehyde linkers [21], as illustrated in Fig 5. Glutaraldehyde is a homo-bifunctional linker and has the potential to bridge two amine functional groups [43]. One end interacts with amine-terminated APTES, and the other end is free to react with the amine-terminated probe DNA. Subsequently, the DNA probe could bind effectively to the silicon nanowire surface to react with the target DNA. The hybridization specificity of the silicon nanowire sensor for the detection of DNA was further evaluated by analyzing fully complementary target DNA and non-complementary target DNA (control group). Fig 9(A) shows the I_ds_-V_ds_ characteristics of silicon nanowire biosensor. The sensor with APTES surface modification on the nanowire surface resulted in I_ds_ = 240 pA, which increased by 131 pA compared with the I_ds_ = 109 pA observed for the initial sensor at V_ds_ = 1 V. Furthermore, upon DNA immobilization and hybridization, a further significant increase in I_ds_ was observed. The sensor with the target complementary DNA (10 μM) resulted in I_ds_ = 857 pA, which is 517 pA higher than the DNA probe (10 μM), I_ds_ = 340 pA at V_ds_ = 1 V. Because the silicon nanowires is p-type, this I_ds_ increment depended on the enhanced hole current density on the surface (due to accumulation of more positive charges carriers) induced by the negatively charged probe/target hybridized DNA, thus resulting in a change in resistance. Fig 9(B) shows that the average resistance of the p-type silicon nanowires decreased upon the addition of negative surface charges at the surface. An obvious 66.5% increase in resistance was observed when 10 μM target complementary DNA was hybridized to the immobilized DNA probe, as shown in Fig 9(C). Consequently, the electrical conductance values of silicon nanowires increased, as shown in Fig 9(D). The increase in conductance for the p-type silicon nanowire device is consistent with an increase in negative surface charge density associated with the binding of negatively charged DNA to the surface. These results are in good agreement with previously reported results [6,21,44]. The multiple layers of surface functionalization (molecules) bound to the silicon nanowires, which caused the current flow to increase, thus proving that the silicon nanowire sensor was sensitive to chemical changes and reactions. To further verify that the current and resistance changes were due to the hybridization of the complementary target DNA, 10 μM of non-complementary target DNA (control group) was hybridized to the immobilized DNA probe. As shown in Fig 9(A), no significant I_ds_ change was observed in this case, which indicates that there is no significant reaction binding of the non-complementary target DNA to the silicon nanowire surface. Furthermore, I_ds_ returned to the original value of the immobilized DNA probe, although there was a negligible resistance change (1.9%) with the non-complementary control group (Fig 9(C)), indicating that no duplex forms between the pair of sequences, which is consistent with the previously reported results [17,19, 23, 44]. Therefore, the silicon nanowires offer very good specificity with excellent discrimination between fully complementary and non-complementary sequences.

**Fig 10:**
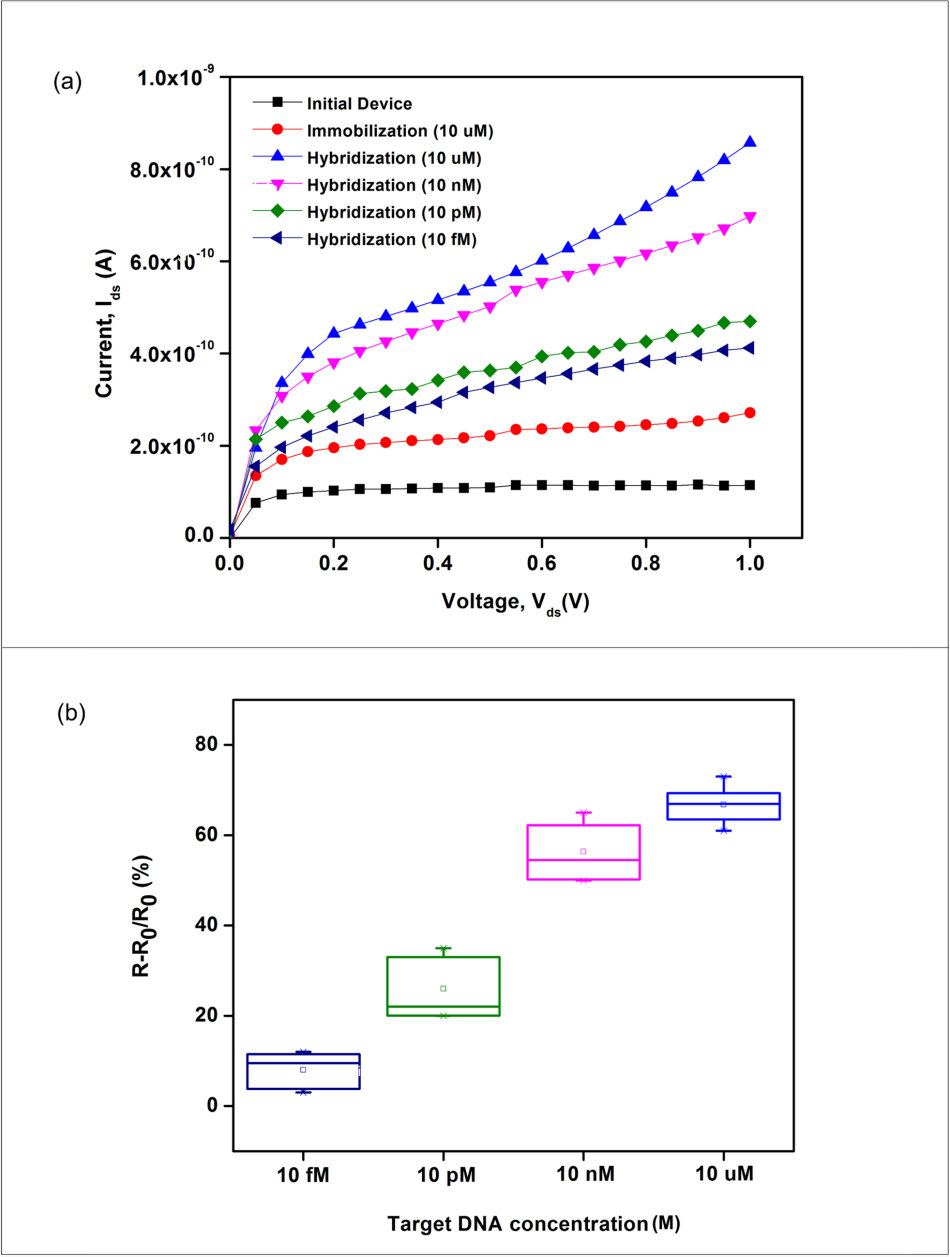
Response of the immobilized DNA silicon nanowires biosensor to the complementary DNA of varying concentrations from 10 μM down to 10 fM. (a) I_ds_-V_ds_ characteristic and (b) resistance changes versus concentration (i.e., relative change in resistance).

DNA detection was further performed by monitoring the concentration-dependent resistance change upon hybridization to complementary target DNA as shown in Fig 10. The relative change in resistance was extracted from the I_ds_-V_ds_ (Fig 10(A)). An obvious 66.5% resistance change was obtained when 10 μM concentration of complementary target DNA was hybridized to the immobilized DNA probe. While, the resistance change reduced to 52.9%, 24.3% and 8.9%, respectively when 10 nM, 10 pM and 10 fM concentration of complementary target DNA were employed. From these results, it can be concluded that, the higher concentration of the target DNA was hybridized, the more negative charges added on the silicon nanowires surface, which lead to an accumulation of more positive charges carriers (holes), resulting in the increasing of the relative change in resistance as observed. Furthermore, this observation was also in consistent with previously reported results [3, 7, 17, 43].

In biosensing, the limit of detection (LOD) is defined as the lowest detectable molar concentration of target DNA, and it is used as a primary measure of sensitivity. In this research, the LOD was evaluated by lowering the concentration of target DNA (10 fM), as shown in Fig 10(B). To the best of our knowledge, the LOD of the p-type silicon nanowire sensor demonstrated in this research is lower than that demonstrated by Pengfei et al. [20], Adam et al. [21], Ryu et al.[44] or Pham et al. [45], which were 1 nM, 0.1 nM, 1 pM and 200 pM, respectively. Our results verify that this silicon nanowire sensor is feasible as a biosensor to detect an extremely low concentration target DNA without additional labeling procedures. Table 3 summarized the analytical performance of our p-type silicon nanowire biosensors in terms of size, detection limit and advantages. Based on the data in Table 3, it is clearly shown that our work has superior performances compared to previous published works. In addition, the potential advantages of using our device are small volume consumption of analytes for biosensing elements, compatibility with integrated circuitry and possibility of multiplexed sensing. Moreover, the fabrication of silicon nanowire by the proposed top-down approach offers an excellent uniformity and reproducibility, and the sensor can be readily incorporated to commercially available detection devices.

**Table 3:** Analytical performance of p-type silicon nanowire in DNA biosensor technologies.

Techniques & Materials		Nanowire width	Lowest detection limit of target DNA	Advantages	Reference
Pengfei	Top-down approach	60 nm -200 nm	1 nM	Higher accuracy chemical and biological molecules detection	[20]
	Anisotropic self-stop etching technique				
	P-type				
	DNA–DNA hybridization				
Adam	Top-down approach	20 nm—1000 nm	0.1 nM	High-sensitive and selective biosensor	[21]
	Trimming using plasma processes				
	P-type				
	DNA–DNA hybridization				
Ryu	Top-down approach	50–80 nm	1 pM	High-sensitive and label-free	[44]
	Gold nanoparticle embedded silicon nanowire				
	P-type				
	DNA–DNA hybridization				
Pham	Top-down approach	40 nm	200 pM	Sensitive and powerful device for bio-detection applications	[45]
	Etching under angle technique				
	P-type				
	DNA–DNA hybridization				
M.Nuzaihan	Top-down approach	20–60 nm	< 10 fM	Ultrasensitive, good specificity and label-free, high uniformity and reproducibility,	Present work
	Size reduction process			Small volume consumption of analytes	
	P-type			Compatible with integrated circuitry and possibility of multiplexed sensing	
	DNA–DNA hybridization				

## Conclusions

High-quality silicon nanowires with good performance as a biosensor were successfully fabricated via a top-down nanofabrication approach. The major challenges in this research involve the EBL process and the reduction of the feature size by thermal oxidation. With the proper manipulation of parameters in the EBL and size-reduction processes, the widths of the silicon nanowires are controllable, decreasing the 70-nm-wide nanowires obtained from the resist pattern to 20-nm-wide and 30-nm-high nanowires with a straight over length of 400 μm. Furthermore, considering the high-quality and repeatable fabrication process of these silicon nanowires, this research shows that silicon nanowires can be fabricated using this approach, which is compatible with established CMOS technologies. The fabricated silicon nanowires showed good electrical characteristics after surface modification, DNA immobilization and DNA hybridization. After the DNA immobilization and hybridization, the current I_ds_ was found to increase, which is consistent with an increase in the negative charge on the nanowire surface due to negatively charged DNA. Thus, by monitoring the resistance and conductance changes upon hybridization to complementary target DNA, the silicon nanowire sensor specifically detects target DNA at femtomolar levels. The use of this approach offer good specificity and sensitivity, with an LOD of 10 fM for target DNA. The fabricated silicon nanowires have great potential as a biosensor for biomedical diagnostic applications.

## References

[1] Shen MY, Li BR, Li YK. Silicon nanowire field-effect-transistor based biosensors: From sensitive to ultra-sensitive. Biosensors and Bioelectronics 2014; 60:101–111. 10.1016/j.bios.2014.03.057 24787124

[2] Hsiao CY, Lin CH, Hung CH, Su CJ, Lo YR, Lee CC, et al Novel polysilicon nanowire field effect transistor for biosensing application. Biosensors and Bioelectronics 2009; 24:1223–1229. 10.1016/j.bios.2008.07.032 18760914

[3] Chen KI, Li BR, Chen Y. Silicon nanowire field-effect transistor-based biosensors for biomedical diagnosis and cellular recording investigation. Nano Today 2011; 6: 131–154.

[4] Cui Y, Wei Q, Park H, Lieber CM. Nanowire Nanosensors for Highly Sensitive and Selective Detection of Biological and Chemical Species. Science 2001; 293: 1289–1292. 11509722 10.1126/science.1062711

[5] Park I, Li Z, Li X, Pisano AP, Williams RS. Towards the silicon nanowire-based sensor for intracellular biochemical detection. Biosensors and Bioelectronics 2007; 22: 2065–2070. 17056246 10.1016/j.bios.2006.09.017

[6] Zheng G, Patolsky F, Cui Y, Wang WU, Lieber CM. Multiplexed electrical detection of cancer markers with nanowire sensor arrays. Nat. Biotechnol., 2005; 23(10): 1294–1301. 16170313 10.1038/nbt1138

[7] Zhang Guo-Jun, Ning Yong, Silicon nanowire biosensor and its applications in disease diagnostics: A review. Analytica Chimica Act 2012; 749: 1–15. 10.1016/j.aca.2012.08.035 23036462

[8] Farahidah Za'bah Nor, Kwa Kelvin SK, Leon Bowen, Budhika Mendis, Anthony O'Neill. Top-down fabrication of single crystal silicon nanowire using optical lithography. Journal of Applied Physics 2012; 112: 024309–024309. ISSN 0021-8979

[9] Vu XT, GhoshMoulick R, Eschermann JF, Stockmann R, Offenhäusser A, Ingebrandt S. Fabrication and application of silicon nanowire transistor arrays for biomolecular detection. Sensors and Actuators B 2010; 144: 354–360

[10] Rius G, Llobet J, Borrisé X, Pérez‐Murano F. Fabrication Of Nanomechanical Devices Integrated In CMOS Circuits By Ion Beam Exposure Of Silicon. AIP Conf. Proc 2011; 1336, 239 10.1063/1.3586095.

[11] Gao A, Lu N, Dai P, Fan C, Wang Y, Li T. Direct ultrasensitive electrical detection of prostate cancer biomarkers with CMOS-compatible n- and p-type silicon nanowire sensor arrays. Nanoscale 2014; 6 (21): 13036–13042. 10.1039/c4nr03210a 25248104

[12] Adam Tijjani, Hashim U, Dhahi ThS, Leow Pei Ling. Nano lab-on-chip systems for biomedical and environmental monitoring. African Journal of Biotechnology 2013; 12(36): 5486–5495.

[13] Shaurya Prakash, Junghoon Yeom. Chapter 5-Lab-on-a-Chip and Fluid Manipulation Applications. Nanofluidics and Microfluidics 2014; In Micro and Nano Technologies: 171–239. ISBN 9781437744699.

[14] Park I, Li Z, Pisano AP, Williams RS. Top-Down Fabricated Silicon Nanowire Sensors for Real-Time Chemical Detection. Nanotechnology, 2010; 21: 1–9. 10.1088/0957-4484/21/1/015501 19946164

[15] Curreli M, Zhang Rui, Ishikawa FN, Hsiao-Kang Chang, Cote RJ, Zhou Chongwu, et al Real-Time, Label-Free Detection of Biological Entities Using Nanowire-Based FETs. IEEE Transactions On Nanotechnology 2008; 7(6): 651–667.

[16] Patolsky Fernando, Zheng Gengfeng, Lieber Charles M. Fabrication of silicon nanowire devices for ultrasensitive, label-free, real-time detection of biological and chemical species. Nature Protocols 2006; 1: 1711–1724. 17487154 10.1038/nprot.2006.227

[17] Gao Z, Agarwal A, Trigg AD, Singh N, Fang C, Tung CH, et al Silicon Nanowire Arrays for Label-Free Detection of DNA. Analytical Chemistry 2007; 79: 3291–3297. 17407259 10.1021/ac061808q

[18] Wenga G, Jacquesa E, Salaüna AC, Rogela R, Pichona L, Genesteb F. Bottom-gate and step-gate Polysilicon nanowires field effect transistors for ultrasensitive label-free biosensing application. Procedia Engineering 2012; 47: 414–417.

[19] Zhang Guo-Jun, Chua Jay Huiyi, Chee Ru-Ern, Agarwal Ajay, She Mein Wong. Label-free direct detection of MiRNAs with silicon nanowire biosensors. Biosensors and Bioelectronics 2009; 24: 2504–2508. 10.1016/j.bios.2008.12.035 19188058

[20] Dai Pengfei, Gao Anran, Lu Na, Li Tie, Wang Yuelin. A Back-Gate Controlled Silicon Nanowire Sensor with Sensitivity Improvement for DNA and pH Detection. Japanese Journal of Applied Physics 2013; 52: 121301.

[21] Tijjani Adam, Hashim U. Highly sensitive silicon nanowire biosensor with novel liquid gate control for detection of specific single-stranded DNA molecules. Biosensors and Bioelectronics 2014; 7184:1–6. 10.1016/j.bios.2014.10.005 25453738

[22] M.Nuzaihan MN, Hashim U, Rahim Ruslinda A, Md Arshad MK, Baharin MHA. Fabrication of Silicon Nanowires Array Using E-beam Lithography Integrated with Microfluidic Channel for pH Sensing. Current Nanoscience, 2015; 11: 239–244.

[23] Zhang GJ, Zhang L, Huang MJ, Luo ZHH, Tay GKI, Lim EJA, et al Silicon nanowire biosensor for highly sensitive and rapid detection of Dengue virus. Sens. Actuators B, 2010; 146: 138–144.

[24] Teo Boon K, Sun XH. From Top-Down to Bottom-Up to Hybrid Nanotechnologies: Road to Nanodevices. Journal of Cluster Science 2006; 17(4): 529–540.

[25] Tran DP, Wolfrum B, Stockmann R, Offenhausser A, Thierry B. Fabrication of locally thinned down silicon nanowires. J. Mater. Chem. C 2014; 2(26): 5229–5234.

[26] Nor MNM, Hashim U, Halim NHA, Hamat NHN. Top down approach: Fabrication of silicon nanowires using scanning electron microscope based electron beam lithography method and inductively coupled plasma-reactive ion etching. Am. Inst. Phys. Conf. Proc., 2010; 1217: 272–278.

[27] Lu Wei, Charles M Lieber: Semiconductor nanowires. J. Phys. D. Appl. Phys. 2006; 39: R387–R406.

[28] Elsner H, Meyer HG. Nanometer and high aspect ratio patterning by electron beam lithography using a simple DUV negative tone resist. Microelectron. Eng. 2000; 57: 291–296.

[29] Voigt A, Elsner H, Meyer HG, Gruetzer G. Nanometer Patterning Using ma-N 2400 Series Duv Negatinve Photoresist and Electron Beam Lithograpy. Proc. SPIE 1999; 3676: 485–491.

[30] Kim Young sang, Jeong Hee jun. Characteristics of negative electron beam resists ma-N2410 and ma-N2405. Microelectronic Engineering 2008; 85: 582–586.

[31] M.Nuzaihan MN, Hashim U, Nazwa T, Adam Tijjani. Resist Mask and Nanowires Formation by Direct-Write Electron Beam Lithography. Journal of Applied Sciences Research 2013; 9(11): 5580–5587.

[32] Goodberlet JG, Hastings JT, Smith HI. Performance of the Raith 150 electron-beam lithography system. J. Vac. Sci. Technol. B Microelectron. Nanom. Struct. 2001; 19, 2499.

[33] Abd Rahman Siti Fatimah, Yusof Nor Azah, Hashim Uda, Nuzaihan M. Nor Md. Design and Fabrication of Silicon Nanowire based Sensor. Int. J. Electrochem. Sci. 2013; 8:10946–10960.

[34] Tseng AA, Chen K, Chen CD, Ma KJ. Electron beam lithography in nanoscale fabrication: Recent development. IEEE Transactions on Electronics Packaging Manufacturing 2003; 26 (2): 141–149.

[35] Kim Y, Jeong H. Characteristics of negative electron beam resists, ma-N2410 and ma-N2405. Microelectronic Engineering, 2008; 85: 582–586.

[36] Grigorescu AE, Van Der Krogt MC, Van Der Drift EWJM, Hagen CW. High dose exposure of silicon in electron beam lithography. Journal of Micro/Nanolithography, MEMS, and MOEMS, 2008; 7 (1), art. no. 013005.

[37] Enami H, Sakaguchi M, Itabashi N, Izawa M. Plasma Etching System and its Applications to 45–32-nm Leading-edge Devices. Hitachi Review 2007; 56(3): 57.

[38] Bonifas AP, McCreery RL, Harris KD. Thermal oxidation as a simple method to increase resolution in nanoimprint lithography. Microelectronic Engineering, 2011; 88(11): 3256–3260.

[39] Nuzaihan M. Nor M, Hashim U, Nazwa T, Adam T. Fabrication of Silicon Nanowires by Electron Beam Lithography and Thermal Oxidation Size Reduction Method. Advanced Materials Research 2014; 832: 415–418.

[40] Dhahi TS, Hashim U, Ahmed NM. Fabrication and Characterization of 50 nm Silicon Nano-Gap Structures. Science of Advanced Materials 2011; 3(2): 233–238.

[41] Za'bah NF, Kwa KS, O'Neill A. The study on the aspect ratio of Atomic Force Microscope (AFM) measurements for Triangular Silicon Nanowire. IEEE Region. Symp. Micro Nano Electron 2013; 223–226.

[42] Wu CC, Ko FH, Yang YS, Hsia DL, Lee BS, Su TS. Label-free biosensing of a gene mutation using a silicon nanowire field-effect transistor. Biosensors and Bioelectronics, 2009; 25 (4): 820–825. 10.1016/j.bios.2009.08.031 19765969

[43] Omair Noor M, Krull Ulrich J. Silicon nanowires as field-effect transducers for biosensor development: A review. Analytica Chimica Acta 2014; 825: 1–25. 10.1016/j.aca.2014.03.016 24767146

[44] Ryu SW, Kim CH, Han JW, Kim CJ, Jung C, Park HG, et al Gold nanoparticle embedded silicon nanowire biosensor for applications of label-free DNAdetection. Biosensors and Bioelectronics 2010; 25 (9): 2182–2185. 10.1016/j.bios.2010.02.010 20227871

[45] Pham VB, Pham XTT, Dang NTD, Le TTT, Tran PD, Nguyen TC, et al Detection of DNA of genetically modified maize by a silicon nanowire field-effect transistor. Advances in Natural Sciences: Nanoscience and Nanotechnology 2011; 2 (2), art. no. 025010,.

